# Dietary habits in women with recurrent idiopathic calcium nephrolithiasis

**DOI:** 10.1186/1479-5876-10-63

**Published:** 2012-03-28

**Authors:** Tiziana Meschi, Antonio Nouvenne, Andrea Ticinesi, Beatrice Prati, Angela Guerra, Franca Allegri, Federica Pigna, Laura Soldati, Giuseppe Vezzoli, Giovanni Gambaro, Fulvio Lauretani, Marcello Maggio, Loris Borghi

**Affiliations:** 1 Department of Clinical Sciences, University of Parma, Via A. Gramsci 14, 43126 Parma, Italy; 2 Department of Medicine, Surgery and Dentistry, University of Milan, Milan, Italy; 3 Nephrology Unit, S. Raffaele Hospital, Milan, Italy; 4 Department of Internal Medicine and Medical Specialties, Renal Program, Columbus-Gemelli University Hospital, Catholic University, Rome, Italy; 5 Geriatric Unit, Parma University Hospital, Parma, Italy; 6 Department of Internal Medicine and Biomedical Sciences, University of Parma, Parma, Italy

**Keywords:** Idiopathic calcium nephrolithiasis, Diet, Kidney stones, Food frequency

## Abstract

**Background:**

Nutrition has been widely recognized to influence the risk of kidney stone formation. Therefore the aim of our study was to assess: a) whether usual diet of women with idiopathic calcium nephrolithiasis (ICN) living in Parma (Northern-Italy) is different compared to healthy controls, b) how their diet differs from Italian National guidelines and c) whether it is related to nephrolithiasis clinical course.

**Methods:**

143 women with recurrent ICN (mean age 43 ± 13 ys) and 170 healthy women (mean age 42 ± 11 ys) were enrolled. All women completed a food frequency questionnaire for the last 60-days and a 3-day dietary diary analysed with a dedicated software.

**Results:**

Stone formers showed a higher consumption of sausages, ham, meat and sweets than healthy controls (43.1% vs 11.1%, 29.4% vs 13.9%, 21.6% vs 4.2%, 66.7% vs 18.1%, p < 0.001). The 3-day diary analysis showed an intake of calories, carbohydrates, lipids and non-discretionary sodium about 10% higher than healthy controls (p < 0.001). Finally, after dividing the population into 3 age groups (≤30, 31-40, > 40 years), the differences described above were amplified in the class ≤30 years, where nephrolithiasis presented a more serious course (shorter recurrence interval, greater stone-rate). In this age group the intake of fruit and vegetables was notably lower than guideline recommendations.

**Conclusions:**

We conclude that the usual diet of women with recurrent ICN is different from controls and characterized by low intake of fruits and vegetables and higher consumption of simple sugars and foods with high protein and salt content. This dietary imbalance could play a role in the ICN pathogenesis, especially in younger women.

This work was financed by grants from Italian Ministry of University and Research as part of a larger project about the prevention of kidney stones (PRIN 2005063822) and by Fondazione per la Ricerca Scientifica Termale (FoRST). No potential conflict of interest relevant to this paper was reported.

## Background

Several evidences in medical literature point out that inadequate nutrition has a direct effect on urinary stone risk factors and on the development of kidney stones [1-6]. A high animal protein diet increases urinary calcium, uric acid, oxalate, and phosphorus and decreases urinary citrate and pH, as an effect of a higher intake of purine and phosphorus and urinary acidification [7-9]. Similar lithogenic changes have been reported with high carbohydrate and fat intake [10-12]. Even salt intake, which is generally much higher than recommended in industrialized countries, represents a powerful factor that increases calciuria and lithogenic risk, in synergy with animal proteins [13-15]. On the other hand, a low calcium intake was formerly thought to be protective against the onset of kidney stones, but nowadays is considered a risk factor, since it promotes the intestinal absorption of oxalate and encourages the onset of bad dietary habits, such as a high animal protein intake [16-18]. Furthermore, a low consumption of fruit and vegetables, leading to an inadequate intake of anti-lithogenic factors such as potassium, magnesium and citrate, is considered a risk factor for kidney stone too, although there are some fruits (such as figs, prunes and raspberries) and vegetables (such as beets, spinach and tomatoes) which are the main dietary sources of oxalate [18-20]. As a matter of fact, the oxalate excretion is poorly related to the dietary intake [21].

A low fluid intake is a heavy risk factor for kidney stone formation, leading to a low urine volume and thus higher urinary concentration of lithogenic substances [22,23]. All kind of drink share this beneficial effect, except few juices (such as apple juice and grapefruit juice), cola and some sport drinks, for their elevated content of oxalate and carbohydrates, such as fructose [24-26].

Although the relationship between diet and kidney stones has been investigated by a large number of studies, results from studies comparing stone former and healthy control dietary habits are contradictory; moreover, whether these supposed differences are important in the development and the progression of the disease is unclear [27-34].

Therefore, in Parma area (Northern Italy), we analyzed the dietary habits of a group of women suffering from recurrent idiopathic calcium nephrolithiasis through two different survey instruments (three-day dietary diary and food frequency questionnaire), comparing them with a healthy control group. We chose to investigate only female subjects because the incidence of kidney stones is rising particularly in women, in a way that seems correlated to dietary changes that occurred in the last decades [18]. Our main purpose was to determine whether the dietary habits of adult female stone formers are significantly different than those of healthy women. Secondly, we aimed to assess whether the food intake of the two groups differ from recommendations of the Italian National Institute for Research on Foods and Nutrition (INRAN) [35]. Finally, we aimed to determine if diet is related to the clinical course of kidney stones in the study sample.

## Materials and methods

At the Kidney Stone Clinic of the Clinical Science Department, University of Parma, we consecutively enrolled 143 normotensive women with recurrent idiopathic calcium nephrolithiasis (mean age 43 ± 13 years). A written informed consent was obtained. The study was performed in compliance with Helsinki declaration. Every patients had previously at least one stone expelled and analyzed, with a stone composition of calcium-oxalate > 80%. No patient had significant co-morbidity (ie, primary hyperparathyroidism, primary hyperoxaluria, inflammatory bowel disease, renal tubular acidosis, sarcoidosis, sponge kidney, hyperthyroidism) or interfering drug therapy capable of modifying the risk of stone risk formation, including biphosphonates, vitamin D, thiazides, calcium supplements, estrogens, alkaline citrate and allopurinol. Patients were defined normotensive according to the European Society of Cardiology and Hypertension guidelines (systolic pressure < 129 mmHg, diastolic pressure < 84 mmHg) [36].

We also enrolled 170 healthy normotensive female volunteers (mean age 42 ± 11 years). For the enrolment of volunteers, different procedures have been used: cooperation with stone formers patients in order to find acquaintances or friends willing and able to take part in the study and targeted invitations to the population residing in Parma. No incentive was given to participants to be enrolled in the present study. The general characteristics of the study subjects are shown in Table 1. All patients have their medical history recorded in computed medical records. A program calculates stone severity through stone rate computation following the formula (total n. of stones - n. of stone at 1^st ^episode/time between 1^st ^and last episode).

**Table 1:** Main characteristics of the women at baseline.

	STONE FORMERS (N = 143)	CONTROLS (N = 170)	p
**AGE ***(yr)*	43 ± 13	42 ± 11	0.32

**WEIGHT ***(kg)*	64 ± 12	62 ± 11	0.32

**HEIGHT ***(cm)*	163 ± 7	163 ± 7	0.51

**BMI ***(Kg/m)^2^*	24,1 ± 4,4	23,5 ± 3,9	0.21

**SYSTOLIC PRESSURE ***(mmHg)*	118 ± 6	118 ± 12	0.11

**DIASTOLIC PRESSURE **(*mmHg*)	76 ± 5	74 ± 8	0.09

**STONE RATE** (number of stones per patient per year)*	0,51 ± 0,76	-	-

**AGE OF THE FIRST EPISODE (yr)**	35,3 ± 14,6	-	-

**INTERVAL BETWEEN** **1ST AND 2^ND ^EPISODE (yr)**	7,1 ± 9,6	-	-

- Data are shown as mean ± standard deviation.

* Stone rate was calculated following the formula (total n. of stones - n. of stone at 1^st ^episode/time between 1^st ^and last episode).

All women in the study, stone formers and controls, filled a dietary diary for three non-consecutive days, reporting all the food intake. This diary was subsequently discussed and interpreted in a visit with a dietician.

The diaries were afterwards analyzed by a specific software with a comprehensive database for a wide variety of macro- and micronutrients (Dietosystem, DS Medica, Milan). This software allows to determine the nutrient composition of every food eaten. We had previously extended the software database in collaboration with the software developers, including recent data from national and international institutions' databases and from scientific papers. We particularly focused on those micronutrients which are notably involved in the pathogenesis of nephrolithiasis, such as sodium, potassium, calcium, oxalate, magnesium and phosphorus.

All stone formers and controls were also given by a dietician a validated food frequency questionnaire of the last 60 days (a facsimile of the form is shown in Additional file 1). This instrument allowed us to perform a further analysis of the dietary habits, focusing on the different intake of milk, dairy products, meat, sausages, ham, fish, eggs, legumes, cereals, fruit, vegetables, sugar, sweets, fats and alcohol.

Data analysis was performed comparing stone formers and controls, and after splicing the whole sample in three subgroups (< 30 years, 31-40 years, > 40 years). In fact, analyzing the clinical history of the patients, it was clear that the subjects under 30 generally show more severe disease (Table 2).

**Table 2:** Clinical course of the disease in relation with age-group.

	Age groups		
	
	< 30 years (mean age 24 ± 5) n. 29	31-40 years (mean age 37 ± 3) n. 38	> 41 years (mean age 53 ± 7) n. 76
**STONE RATE** *number of stones per patient per year#*	1,2 ± 1, 2*	0,7 ± 0,8	0,4 ± 0,5

**AGE AT 1^st ^EPISODE** *(years)*	21 ± 6*	30 ± 8	42 ± 15

**INTERVAL BETWEEN 1ST AND 2ND EPISODE** *(years)*	2,4 ± 2,2*	5 ± 5,8	7,5 ± 8,4

- Data are shown as mean ± standard deviation.

* = p < 0.01 vs other groups.

#Stone rate was calculated following the formula (total n. of stones - n. of stone at 1^st ^episode/time between 1^st ^and last episode).

Results were analyzed using suitable statistical tests: the frequencies were compared using the χ^2 ^Fisher test, while the data of the bromatological decomposition were reported as mean ± standard deviation (SD) and compared using Student T test (SPSS, Chicago, IL).

Agreement of Unique Ethical Committee of Province of Parma, Italy, was obtained.

## Results

No differences in the main characteristics of stone formers and controls were found (Table 1).

Dividing stone formers into three age subgroups (under 30 years, 31-40 years, over 40 years), women under 30 showed a more severe disease (earlier age of onset, shorter recurrence interval, higher stone rate) (Table 2).

The analysis of food intake by bromatological separation of the three-day dietary diary showed that female stone formers have a calorie, carbohydrate, protein, sodium and magnesium intake significantly higher than the control group (Table 3). There were no differences in water, fat, fiber, potassium, calcium, oxalate, and phosphorus intake. Moreover, dividing the sample into three age subgroups (under 30 years, 31-40 years, over 40 years), also a lower intake of water (950 ± 500 vs 1730 ± 710 ml, p = 0.0003) and a higher intake of lipids (83 ± 34 vs 70 ± 22 ml, p = 0.05) were found in the stone formers under 30 years.(Table 3)

**Table 3:** Comparison between bromatologic decomposition (dietary intake) of the three-non-consecutive day dietary diaries of stone formers and healthy controls.

	STONE FORMERS (SF) (N = 143)	CONTROLS (CTRL) (N = 170)	p	SF	CTRL	p	SF	CTRL	p	SF	CTRL	p
									
				< 30 ys	< 30 ys		31-40 ys	31-40 ys		> 41 ys	> 41 ys	
									
				(N = 29)	(N = 33)		(N = 38)	(N = 46)		(N = 76)	(N = 91)	
**H_2_O ***(ml)*	1970 ± 679	1942 ± 685	0,5	950 ± 500	1730 ± 710	**0,0003**	1998 ± 582	1915 ± 445	0.5	2034 ± 592	2006 ± 561	0.7

**Caloric intake ***(Kcal)*	2116 ± 1105	1944 ± 724	**0,004**	2138 ± 673	1790 ± 466	**0,01**	2225 ± 855	1793 ± 454	**0,01**	2140 ± 773	1938 ± 557	**0.02**

**Carbohydrates ***(g)*	271 ± 185	241 ± 92	**0,001**	290 ± 77	252 ± 71	**0.04**	301 ± 177	220 ± 61	**0.02**	262 ± 92	240 ± 69	**0.0006**

**Proteins ***(g)*	79 ± 35	75 ± 26	**0,04**	85 ± 26	70 ± 17	**0,005**	82 ± 26	70 ± 19	**0.03**	79 ± 18	75 ± 28	**0.05**

**Lipids ***(g)*	82 ± 54	76 ± 49	0,07	83 ± 34	70 ± 22	**0.05**	83 ± 19	77 ± 16	0.2	80 ± 43	80 ± 38	0.9

**Fibers ***(g)*	15,6 ± 11,5	15,1 ± 7,9	0,4	12,1 ± 5,4	14,1 ± 6,3	0.2	15,7 ± 9,9	12,4 ± 4,5	0.09	16,5 ± 7,2	16,3 ± 5,7	0.9

**Sodium ***(mg)*	2149 ± 2394	1683 ± 1196	**< 0,0001**	2551 ± 2374	1433 ± 575	**0.004**	2467 ± 1640	1729 ± 1075	**0.04**	2037 ± 781	1791 ± 793	**0.05**

**Potassium ***(mg)*	2454 ± 1085	2421 ± 914	0,6	2181 ± 764	2208 ± 668	0.9	2499 ± 742	2239 ± 556	0.1	2529 ± 878	2596 ± 672	0.6

**Calcium ***(mg)*	738 ± 513	728 ± 429	0,7	680 ± 360	717 ± 271	0.7	707 ± 294	682 ± 221	0.4	766 ± 390	742 ± 332	0.7

**Oxalate ***(mg)*	141 ± 209	127 ± 146	0,2	126 ± 94	126 ± 83	0.7	126 ± 70	112 ± 65	0.2	155 ± 182	134 ± 99	0.4

**Magnesium ***(mg)*	269 ± 144	247 ± 119	**0,01**	259 ± 94	218 ± 63	**0.03**	281 ± 116	235 ± 64	**0.05**	267 ± 81	260 ± 88	0.6

**Phosphorus ***(mg)*	1049 ± 435	1013 ± 416	0,2	1007 ± 411	964 ± 262	0.6	1017 ± 226	963 ± 324	0.4	1067 ± 324	1045 ± 315	0.7

Data are shown as daily mean ± standard deviation.

Using the food frequency tool, we also found that stone formers have a significantly higher consumption of sausages, ham, meat and sweets than the controls (Table 4, Additional file 2).

**Table 4:** Results from food frequency questionnaire in all stone formers and controls.

PERCENTAGE OF WOMEN WITH INAPPROPRIATE INTAKE (VARIATIONS FROM RECOMMENDATIONS) OF VARIOUS NUTRIENTS ACCORDING TO INRAN GUIDELINES			
	**STONE** **FORMERS** **n. 143**	**CONTROLS** **n. 170**	**p**

SAUSAGES* in excess (> 3 portions/week)	43,1%	11,1%	**< 0,0001**

**HAM§** *in excess *(> 3 portions/week)	29,4%	13,9%	**< 0,0001**

**SWEETS#** *in excess *(> 1 portion/day)	66,7%	18,1%	**< 0,0001**

**MEAT (**excluding sausages and ham) *in excess *(> 1 portion/day)	21,6%	4,2%	**< 0,0001**

The portions for each food are given in Additional file 2.

*Sausages: Italian Typical Foods (*Salame, Coppa, Mortadella, Pancetta*)

§Ham: Italian Typical Foods *(Parma Ham, Baked Ham, Culatello, Fiocchetto, Spalla)*

#Sweets: cakes, biscuits, pastries, simple sugar, ice cream, toffees

Finally, after dividing the sample into the three age subgroups, we found that the differences described above are amplified in the subjects under 30: in fact in female stone formers under 30 years we found a greater intake of sausages, ham and meat and a lower intake of fruit and vegetables, with high statistical significance (Table 5).

**Table 5:** Results from food frequency questionnaire in stone formers and controls under 30.

PERCENTAGE OF UNDER 30 WOMEN WITH INAPPROPRIATE INTAKE (VARIATIONS FROM RECOMMENDATIONS) OF VARIOUS NUTRIENTS ACCORDING TO INRAN GUIDELINES			
	**STONE FORMERS** **< 30 ys** **n. 29**	**CONTROLS** **< 30 ys** **n. 39**	**p**

SAUSAGES* in excess (> 3 portions/week)	41,2%	7,7%	**< 0,0001**

**HAM§** *in excess *(> 3 portions/week)	35,3%	10,3%	**< 0,0001**

**MEAT (**excluding sausages and ham) *in excess *(> 1 portion/day)	41,2%	2,6%	**< 0,0001**

**FRUIT AND** **VEGETABLES** *Lacking *(< 5 portions/day)	58,8%	23,1%	**< 0,0001**

The portions for each food are given in Additional file 2.

*Sausages: Italian Typical Foods (*Salame, Coppa, Mortadella, Pancetta*)

§Ham: Italian Typical Foods *(Parma Ham, Baked Ham, Culatello, Fiocchetto, Spalla)*

#Sweets: cakes, biscuits, pastries, simple sugar, ice cream, toffees

We also found that a significant percentage of women, even in the control group, have dietary habits that are consistently different than those recommended by INRAN (Table 4).

## Discussion

The role of diet in the pathogenesis of idiopathic calcium nephrolithiasis has long been investigated. In spite of this, there is no wide consensus about which wrong dietary habits are critical in this process. Our findings seem to suggest that a high intake of calories, carbohydrates, proteins and salt are the main dietary factors associated with calcium nephrolithiasis in women. These findings overlap with those previously reported by Trinchieri et al [32], who also demonstrated that people with recurrent calcium nephrolithiasis have a higher dietary intake of purines and fats. However, those results were related to a mixed male-female cohort, and were not confirmed considering only the female part of the cohort. Moreover, a large number of older studies failed to demonstrate a positive correlation between carbohydrate and protein intake and idiopathic calcium nephrolithiasis [27-31], revealing in some cases a positive correlation with fat intake [27].

A more recent study by Al Zahrani et al [34] showed a positive correlation between calcium nephrolithiasis and calorie and carbohydrate intake, but not with protein intake. It also pointed out that stone formers have an excessive intake of dietary sodium, another element coherent with our findings. The relation between salt and nephrolithiasis has been studied and understood only in the last decade, and some interventional studies have demonstrated that lowering salt intake may lead to a reduced risk for kidney stone onset or recurrence [37-39].

We did not find differences in the intake of calcium between stone formers and controls. However, a low dietary intake of calcium is a well known risk factor for calcium nephrolithiasis, as it leads to an increase in oxalate intestinal absorption [20]. Some studies actually demonstrated that recurrent idiopathic calcium nephrolithiasis patients have a lower intake of calcium than healthy controls [33,34]. It is possible that nowadays stone recurrent patients are aware that a low-calcium diet is not suitable for their disease, and therefore do not limit the intake of milk and dairy products, as they did in the past. Also oxalate intake was not different in the two groups; however we could not estimate urinary oxalate which could be higher in stone formers: in fact carbohydrates, protein and salt intake was significantly higher in our stone formers group and it is well-known that an increased assumption of some sugars (i.e. lactose, sucrose, fructose, xylitol and sorbitol), some aminoacids (i.e. glycine, tyrosine, tryptophan, phenylalanine and hydroxyproline), and salt can augment the oxalate endogenous production and urinary oxalate excretion [25,38-42]. Unfortunately, we cannot estimate in our group dietary habits before stone appearance nor we can estimate what dietary advice the women studied have received before this study. Moreover, it is obvious that the risk of kidney stone recurrence is not determined by a single or by few dietary inbalances, but is a multi-factorial process determined by the sum of a large number of risk factors, all of whom are not necessarily present at the same time.

The role of diet in the pathogenesis of recurrent calcium nephrolithiasis is therefore outstanding. Indirect proof comes also from the perspective studies published in the last years [37-41,43]. These studies show that a change of dietary habits may decrease kidney stone occurrence. In particular, the limitation of salt and animal protein intake, with a normal intake of calcium, may adequately prevent kidney stones [37-41] but may not prevent stone recurrence [43].

Moreover, according to recent data, the salt, carbohydrate and protein excess and the deficiency of fruit and vegetables could increase the stone risk not only through a direct action on the urinary composition, but also through a systemic action, determining changes in the acid-base balance, particularly in the intracellular compartment; Frassetto et al [15] demonstrated that American diet induces a tonic baseline low-grade metabolic acidosis for an imbalance in the supply of nutrient precursors of bicarbonate and hydrogen ions, and kidney mitigates, but does not reduce to zero, the severity of this diet-induced acidemia and hypobicarbonatemia. Some studies confirm this hypothesis, by demonstrating that the urinary composition of stone formers and controls is not different for a large number of substances, even if there are wide differences in the nutrient intake [31]. Only urinary calcium is constantly higher in stone formers in all studies [3,31-34].

Hence, a diet rich in proteins, carbohydrates and sodium is also a diet with a high acid load because it leads to a decrease in the intake of the bicarbonate precursors (such as citrate). It also determines a low potassium intake and it increases the endogenous genesis of H + ions through energetic cycles (i.e. glycolisis, proteolysis, lipolysis). This exposes the body to a state of chronic low-grade acidosis. Therefore, there is an activation of homeostatic mechanisms, involving both the kidney and the body reserves of alkaline load, such as connective tissue and bone [44]. The buffering activity of bone, resulting from the activation of osteoclasts and the increase of the bone reabsorption, may contribute to explain the significant prevalence of hypercalciuria in stone formers compared to healthy controls [45]. This pathophysiological hypothesis seems also plausible in our patients.

A peculiarity of our research is the simultaneous use of two nutritional analysis tools in the same subjects: the bromatological food decomposition recorded by 3-day diary and the food frequency questionnaire. This method has been reported in literature only once [31].

The food frequency questionnaire showed data that are coherent with the findings obtained through the 3-day dietary diary: a high percentage of female stone formers has a consumption of sausages, ham, meat and sweets (foods rich in carbohydrates, proteins and salt) higher than that recommended in the INRAN guidelines. We must point out that the dietary differences between stone formers and controls seem much wider when recorded through the food frequency questionnaire than through the 3-day dietary diary. These findings suggest that the assessment of dietary habits over an extended period of time is a more sensitive tool than the methodical collection of dietary intake for a short period. In fact, while the dietary record by food diary has certainly some advantages, such as the precise determination of the quantities of every food consumed and the possibility of a bromatological decomposition, it also has consistent limits, such as the possible onset of Hawthorne effect (phenomenon in which the awareness of being under observation causes a change in a behaviour or habit), the short length of the record and the possibility of bias due to the software that determines the composition of different foods. This updated software is indeed influenced by the variations of dosage of the chemical system and of food processing. On the other hand, the record by food frequency, being retrospective and conducted on longer periods of time, provides a more precise picture of the real eating habits of the subject, even if it is less specific in determining the quantity of every food eaten [46]. Thus, we believe that using two different tools for recording eating habits is preferable to choosing one or another.

Our results also highlight a feature which is uncommon in literature: the main nutritional imbalances, and a more severe disease course as well (earlier age of onset, shorter recurrence interval, higher stone rate), are characteristic of women under 30. These subjects seem to be more prone to develop nephrolithiasis, but they are also predisposed to suffer from hypertension, atherosclerosis, diabetes and metabolic syndrome, all chronic illnesses which are strongly influenced by unhealthy dietary habits.

The main surveys published in literature, even the prospective ones, followed patients and controls aged between 40 and 75 years old [7]. Within the limits of our little sample, the results we obtained in the younger cohort represent a stimulus to carry out research in this age group. Moreover, our findings represents the dietary habits of women in Northern Italy and they could not be extended to other countries; therefore the supposed pathogenic role of an imbalanced diet can be confirmed only by a broader and perspective research, together with an intervention study.

## Conclusions

In this paper we showed that the dietary habits in women with recurrent idiopathic calcium nephrolithiasis are significantly different than those of healthy controls. These habits consist in a low fruit and vegetable intake and in a high intake of simple sugars, protein and salt. This nutritional imbalance is more evident in younger subjects and in patients with a more severe disease who showed moreover a lower fluid intake and a higher intake of fats. We hope that wide awareness campaigns will rise to reduce wrong dietary habits, that are well known risk factors not only for nephrolithiasis but also for other widespread disease such as diabetes, hypertension, metabolic syndrome and osteoporosis.

## Competing interests

The authors declare that they have no competing interests.

## Authors' contributions

TM, LB, LS, GV and GG designed research; TM, AN, BP, FP and FA conducted research; AG, FL and AT analysed data; TM, AN, AT and MM wrote the paper; AN had primary responsibility for final content. All authors read and approved the final manuscript.

## Supplementary Material

Additional file 1 **Facsimile of the food frequency questionnaire form used in our research**. Click here for file

Additional file 2 **Standard portions in Italian Diet according to the INRAN guidelines**. Click here for file
