# Supplementary material for: Dietary habits in women with recurrent idiopathic calcium nephrolithiasis
Source: J Transl Med. 2012 Mar 28;10:63. doi: 10.1186/1479-5876-10-63 (PMC3337252; doi:10.1186/1479-5876-10-63)
Supplement: Additional file 1 — Facsimile of the food frequency questionnaire form used in our research. [file 1479-5876-10-63-S1.PDF]

Supplemental file 1 – Facsimile of the food frequency questionnaire form used in our research.

FOOD FREQUENCY QUESTIONNAIRE

Name ___________________ Surname ______________________

Sex Male Female

Address ____

Telephone Number ____

Place of Birth _________________ Date of Birth _____________

Age ______ Weight ___________ Height ___________

Date of filling ___________ ID _____________________

DRINKS												
	PORTION			FREQUENCY (every…)			NUMBER OF TIMES					
	A	B	C	day	week	month	1	2	3	4	5	6
Coffee												
Tea												
Cappuccino												
Full-cream milk												
Partially skimmed milk												
Nonfat milk												
Full-cream yoghurt<br>natural<br>fruit<br>cereal												
Nonfat yoghurt<br>natural<br>fruit<br>cereal												
Sugar												
Sparkling drinks<br>orange<br>bitter orange<br>cola<br>others												
Juices<br>orange<br>grapefruit<br>apple<br>pear<br>peach<br>apricot												

FIRST COURSES

	PORTION			FREQUENCY (every…)			NUMBER OF TIMES					
	A	B	C	day	week	month	1	2	3	4	5	6
Pasta with tomato sauce												
Pasta with other vegetable sauce												
Pasta with olive oil												
Pasta with butter												
Pasta with ragout												
Pasta with legumes												
Minestrone (soup)<br>with pasta<br>with rice												
Pureed vegetables												
Meat broth<br>with bread<br>with pasta<br>with rice												
Tortellini and ravioli<br>with ragout<br>with tomato sauce<br>with butter/oil												
Lasagne												
Cannelloni												
Risotto<br>with ragout<br>with tomato sauce<br>with butter/oil												
Polenta (mush)												
Gnocchi (potato dumplings)												

BAKERIES, CEREALS AND SWEETS												
	**PORTION**			**FREQUENCY (every…)**			**NUMBER OF TIMES**					
	**A**	**B**	**C**	**day**	**week**	**month**	**1**	**2**	**3**	**4**	**5**	**6**
White bread												
Wholewheat bread												
Breadsticks, crackers												
Rusks												
Pizza<br>takeaway<br>pizzeria												
Biscuits / Crispbreads												
Cream biscuits / Wafers												
Butter / Egg biscuits												
Croissant / Cornetto												
Cakes / Tarts / Pies												
Pastries												
Corn / Oat flakes												
Puffed rice												

MEATS												
	PORTION			FREQUENCY (every…)			NUMBER OF TIMES					
	A	B	C	day	week	month	1	2	3	4	5	6
Steaks or hamburgers<br>beef<br>pork												
Boiled meat<br>braised<br>stew<br>spezzatino												
Liver and other guts												
Rabbit												
Chicken												
Turkey												
Cooked meats<br>sausages<br>salame<br>mortadella<br>capocollo<br>coppa<br>wurstel												
Bresaola (dried beef)												
Ham (any kind)												
Speck ham												
Bacon												

EGGS												
	PORTION			FREQUENCY (every…)			NUMBER OF TIMES					
	A	B	C	day	week	month	1	2	3	4	5	6
Omelette												
Hard-boiled eggs, fried eggs												

FISH												
	**PORTION**			**FREQUENCY (every…)**			**NUMBER OF TIMES**					
	**A**	**B**	**C**	**day**	**week**	**month**	**1**	**2**	**3**	**4**	**5**	**6**
Blue fish<br>anchovies<br>pilchards<br>mackerels												
Other sea and lake fish<br>codfish<br>sea bass<br>sea bream<br>stockfish<br>dogfish<br>sole<br>mullet<br>trout<br>other _____________												
Squid, devilfish, cuttlefish												
Mixed fried fish												
Shellfish<br>lobster<br>shrimps<br>mussels<br>oysters<br>clams												
Canned fish<br>tuna<br>pilchards<br>mackerels												

CHEESE AND DAIRY PRODUCTS												
	**PORTION**			**FREQUENCY (every…)**			**NUMBER OF TIMES**					
	**A**	**B**	**C**	**day**	**week**	**month**	**1**	**2**	**3**	**4**	**5**	**6**
Light soft cheese<br>ricotta cheese<br>cottage cheese<br>others												
Fat cheese<br>caciotta<br>goat’s cheese<br>certosa cheese<br>fior di latte<br>quark cheese<br>mozzarella<br>parmigiano<br>sheep ricotta cheese<br>scamorza<br>stracchino												
Ripe cheese<br>bel paese<br>brie<br>ripe caciotta<br>caciocavallo<br>camembert<br>cheddar<br>emmenthal<br>fontina<br>processed cheese<br>gorgonzola<br>Gruyère<br>pecorino<br>provolone<br>quartirolo<br>robiola<br>taleggio												

SNACKS												
	**PORTION**			**FREQUENCY (every…)**			**NUMBER OF TIMES**					
	**A**	**B**	**C**	**day**	**week**	**month**	**1**	**2**	**3**	**4**	**5**	**6**
Sweet snacks												
Salty snacks												

VEGETABLES												
	**PORTION**			**FREQUENCY (every…)**			**NUMBER OF TIMES**					
	**A**	**B**	**C**	**day**	**week**	**month**	**1**	**2**	**3**	**4**	**5**	**6**
Green salad<br>lettuce<br>radicchio<br>endive<br>rocket												
Raw vegetables<br>carrots<br>peppers<br>tomatoes<br>artichoke<br>cucumber<br>fennel<br>mushrooms<br>celery												
Cooked vegetables<br>Swiss chard<br>chicory<br>spinach<br>broccoli<br>cauliflower<br>cabbage<br>artichoke<br>green bean<br>fennel<br>aubergine<br>courgette<br>carrots<br>peppers<br>tomatoes												
Boiled or mashed potatoes												
Fried potatoes												
Beans, chickpeas, lentils												
Peas												

FRUITS												
	**PORTION**			**FREQUENCY (every…)**			**NUMBER OF TIMES**					
	**A**	**B**	**C**	**day**	**week**	**month**	**1**	**2**	**3**	**4**	**5**	**6**
Apple, pear												
Banana												
Orange												
Tangerine												
Grapefruit												
Kiwi												
Other seasonal fruit<br>apricot<br>cherries<br>watermelon<br>strawberries<br>melon<br>peach<br>plum<br>grapes												
Walnuts, hazelnuts, almonds												

OTHER FOOD												
	PORTION			FREQUENCY (every…)			NUMBER OF TIMES					
	A	B	C	day	week	month	1	2	3	4	5	6
Fruit in syrup												
Ice-creams												
Ice lollies												
Jam												
Honey												
Chocolate												
Candies												

ALCOHOLIC DRINKS												
	**PORTION**			**FREQUENCY (every…)**			**NUMBER OF TIMES**					
	**A**	**B**	**C**	**day**	**week**	**month**	**1**	**2**	**3**	**4**	**5**	**6**
Wine												
Beer												
Spirits<br>bitters<br>grappa (Italian brandy)<br>cognac<br>liqueur<br>whisky												
Coffee laced with spirits												

When do you usually have alcoholic drinks? **On meals**
Out of meals
Both

In what days do you usually take alcoholic drinks? **In work days**
In weekends
Both
