# Supplementary material for: Dietary habits in women with recurrent idiopathic calcium nephrolithiasis
Source: J Transl Med. 2012 Mar 28;10:63. doi: 10.1186/1479-5876-10-63 (PMC3337252; doi:10.1186/1479-5876-10-63)
Supplement: Additional file 2 — Standard portions in Italian Diet according to the INRAN guidelines. [file 1479-5876-10-63-S2.PDF]

Supplemental file 2 - Standard portions in Italian Diet according to the INRAN guidelines.

Type of food	Foods	Portion	Weight (g)
CEREAL and TUBERS	Bread	Roll, slice	50
	Bakery	2-4 biscuits, 2,5 rusks	20
	Pasta or rice	1 medium portion	80
	Noodles	1 small portion	120
	Potatoes	2 small potatoes	200
VEGETABLES and FRUITS	Salad	1 medium portion	50
	Vegetables	1 fennel, 2 artichokes	250
	Fruits or juice	1 medium fruit (apple)	150
		2 small fruits (apricot)	150
MEAT, FISH, EGGS, PULSES	Fresh meat	1 small slice	70
	Sausages	3-4 medium slices	50
	Fish	1 small portion	100
	Eggs	1 egg	60
	Dried pulses	1 medium portion	30
	Fresh pulses	1 medium portion	80-120
MILK and DERIVED	Milk	1 cup	125
	Yoghurt	1 pot	125
	Fresh cheese	1 medium portion	100
	Matured cheese	1 medium portion	50
FATS	Oil	1 spoon	10
	Butter	1 portion	10
	Margarine	1 portion	10

From: Italian Guidelines for an healthy Italian Diet pag. 71– Italian National Institute for Research in Foods and Nutrition –. INRAN http://www.inran.it/648/linee_guida.html.
